# Phytochemical Profiles, Antioxidant, Cytotoxic, and Anti-Inflammatory Activities of Traditional Medicinal Plants: *Centaurea pichleri* subsp. *pichleri*, *Conyza canadensis*, and *Jasminum fruticans*

**DOI:** 10.3390/molecules27238249

**Published:** 2022-11-26

**Authors:** Derya Çiçek Polat, Selen İlgün, Gökçe Şeker Karatoprak, Esra Küpeli Akkol, Raffaele Capasso

**Affiliations:** 1 Department of Pharmaceutical Botany, Faculty of Pharmacy, Ankara University, Ankara 06560, Turkey; 2 Department of Pharmaceutical Botany, Faculty of Pharmacy, Erciyes University, Kayseri 38039, Turkey; 3 Department of Pharmacognosy, Faculty of Pharmacy, Erciyes University, Kayseri 38039, Turkey; 4 Department of Pharmacognosy, Faculty of Pharmacy, Gazi University, Etiler, Ankara 06330, Turkey; 5 Department of Agricultural Sciences, University of Naples Federico II, Via Università 100, 80055 Portici, Italy

**Keywords:** *Centaurea* sp., *Conyza* sp., *Jasminum* sp., antioxidant, anti-inflammatory, cytotoxic activity, phenolics

## Abstract

*Centaurea pichleri* subsp. *pichleri*, *Conyza canadensis*, and *Jasminum fruticans* are traditionally used plants grown in Turkey. Methanol extracts were obtained from these plants and pharmacological activity studies and phytochemical analyses were carried out. To evaluate the phytochemical composition, spectrophotometric and chromatographic techniques were used. The extracts were evaluated for antioxidant activity by DPPH^●^, ABTS^●+^ radical scavenging, and FRAP assays. The cytotoxic effects of the extracts were investigated on DU145 prostate cancer and A549 lung cancer cell lines. The anti-inflammatory effects of extracts were investigated on the NO amount, TNF-α, IFN-γ, and PGE 2 levels in lipopolysaccharide-stimulated Raw 264.7 cells. The richest extract in terms of phenolic compounds (98.19 ± 1.64 mg_GAE_/g_extract_) and total flavonoids (21.85 ± 0.64 mg_CA_/g_extract_) was identified as *C. pichleri* subsp. *pichleri* methanol extract. According to antioxidant activity determinations, the *C. pichleri* subsp. *pichleri* extract was found to be the most active extract. Finally, the *C. pichleri* subsp. *pichleri* methanol extract was revealed to be the most effective inhibitor of viability in the cytotoxic activity investigation, and the extract with the best anti-inflammatory action. The findings point to *C. pichleri* subsp. *pichleri* as a promising source of bioactive compounds in the transition from natural sources to industrial uses, such as new medications, cosmeceuticals, and nutraceuticals.

## 1. Introduction

From the past to the present, phenolic compounds have been extensively studied for their ability to counteract oxidative stress in chronic oxidative stress, various metabolic disorders, and conditions of obesity, diabetes, renal and cardiovascular disease. When the overproduction of reactive oxygen species (ROS) exceeds the natural antioxidant system (enzymatic and non-enzymatic) defenses, oxidative stress occurs, which damages cellular and extracellular macromolecules. The antioxidant power of phenolic compounds is related to the reducing properties of free radical scavengers as hydrogen or electron-donating agents. In addition, due to the molecular structure of phenolic compounds, they suppress the metal-catalyzed free radical formation and can chelate metals, especially iron and copper [1].

Phenolic compounds have an important place in human health due to their bioactive effects, such as anti-aging, anticancer, anti-inflammatory, cardio-protective, neuroprotective, immunomodulatory, antidiabetic, antibacterial, antiparasitic, and antiviral properties [2]. Therefore, recent studies, especially the evaluation of traditionally used plants in terms of biological activities and the discovery of their active compounds have an important place [3].

Turkey is the main diversity center for *Centaurea* L. species belonging to the Asteraceae family. This genus is represented in Turkey by approximately 226 species [4]. Many Centaurea species have traditionally been used for the treatment of diabetes, diarrhea, hypertension, malaria, microbial infections, rheumatism, wound healing, and ulcers [5,6,7]. *Centaurea pichleri* Boiss. subsp. *pichleri* is known as “gelin düğmesi, peygamber çiçeği or düğmeli ot” in Turkey [8,9]. Although there is not much phytochemical research on these species, studies that assess essential oil and fatty acid content stand out [9].

*Conyza canadensis* (L.) Cronquist (syn. *Erigeron canadense* L.) is an annual herb belonging to the Asteraceae family, known as horseweed or Canadian fleabane [10]. It is a plant of North American origin, but in Europe, it is considered a completely naturalized herb [11]. It is mentioned as it is an invasive species, known as “selviotu, bit out, or Kanada şifaotu” in Turkey. This herb has traditionally been used as a diuretic, to treat constipation, and a decoction of its aerial parts has been used to treat lice [5,8,12]. Additionally, it is well-known that *C. canadensis* is employed in conventional therapy to treat gastrointestinal issues as well as rheumatic symptoms [13]. 

The *Jasminum* L. (Oleaceae) genus has about 200 species and grows in temperate climate regions. Some *Jasminum* species are valued in perfumery and garden plants because of their fragrance [14]. In Turkey, *Jasminum fruticans* L. grows naturally and is known as “sarı yasemin, yaban yasemini, öküzgözü or boruk”. In traditional uses, the flower infusion (5%) is used as a diuretic and for anthelmintic effect in Turkey [5,15]. In other countries, young flowering branches and leaves are traditionally used as a remedy for parasitic diseases, diabetes, allergies, and fatigue [16,17,18,19,20].

Owing to the therapeutic relevance of plants, the current study was designed to assess the phytochemical composition and antioxidant, cytotoxic, and anti-inflammatory potential of the extracts of *Centaurea pichleri* subsp. *pichleri, Conyza canadensis*, and *Jasminum fruticans*. Methanolic extracts of the plants were measured for their phenolic and flavonoid content, while HPLC studies were performed to reveal the secondary metabolic picture. The antioxidant capability of the extracts was measured using a variety of bioassays (DPPH, ABTS, FRAP), and the cytotoxicity of each extract against the DU145 and A549 cell lines was also assessed. Since the anti-inflammatory activities of the species have not yet been identified, inflammation-related NO, TNF-α, IFN-γ, and PGE 2 levels were evaluated in Raw 264.7 cells (mouse macrophage cell line). This is the first report to combine both the pharmacological activity potential and the phytochemical composition of these plants used in traditional medicine.

## 2. Results and Discussion

### 2.1. Chemical Analysis

Herbal extracts high in phenols and flavonoids boost human health by preventing and curing a variety of ailments [3]. Considering these findings, as well as the traditional uses of *Centaurea pichleri* subsp. *pichleri*, *Conyza canadensis*, and *Jasminum fruticans*, we studied the phenolic and flavonoid contents of their methanolic extracts. The total phenol and total flavonoid values for the three plant extracts are given in Table 1. 

The *C. pichleri* subsp. *pichleri* methanol extract was found to have the highest total phenol (98.19 ± 1.64 mg_GAE_/g_extract_) and total flavonoid (21.85 ± 0.64 mg_CA_/g_extract_) amounts (Table 1); although no phytochemical studies on *C. pichleri* subsp. *pichleri* have been conducted, and investigations on other species’ total phenol and flavonoid contents have been performed. 

In the study conducted with eight *Centaurea* species (*C. calolepis* Boiss., *C. cariensis* Boiss. subsp. *maculiceps* (O. Schwarz) Wagenitz, *C. cariensis* Boiss. subsp. *microlepis* (Boiss) Wagenitz, *C. hierapolitana* Boiss, *C. cadmea* Boiss, *C. depressa* Bieb., *C. urvillei* DC. subsp. *urvillei* and *C. ensiformis* P.H. Davis), n-hexane, chloroform and methanol extracts of the species were prepared. Among the n-hexane extracts, the highest total phenol content was found in the hexane extract of *C. urvillei* subsp. *urvillei* (42.11 ± 2.01 mg/L), the highest total phenol amount was found in the chloroform extract of *C. calolepis* (72.11 ± 3.10 mg/L) among the chloroform extracts, and the highest total phenol content was found in the methanol extract of *C. cariensis* subsp. *maculiceps* (120.90 ± 10.90 mg/L) among the methanol extracts [21]. In another investigation, the maximum total phenol concentration was discovered in *C. ptosimopappa* Hayek among both water (120.50 ± 1.66 mg _GAE_/g) and methanol (72.63 ± 1.84 mg _GAE_/g) extracts of three distinct Centaurea species (*C. calcitrapa* L. subsp. *calcitrapa*, *C. spicata* Boiss., and *C. ptosimopappa* Hayek) [22]. In a study by Zengin et al. (2016), the total phenolic and total flavonoid content of eight different *Centaurea* species (*C. depressa* M. Bieb.; *C. drabifolia* subsp. *detonsa* (Bornm.) Wagenitz; *C. kotschyi* var. *persica* (Bornm.) Wagenitz; *C. patula* Bornm.; *C. pulchella* Ledeb.; *C. tchihatcheffi* Fisch. and C.A. Mey.; *C. triumfettii* All.; *C. urvillei* DC subsp. *hayekiana*) were determined by using both ethyl acetate and chloroform extracts. According to the results, the highest total phenol content was found in the chloroform extract of *C. pulchella* (119.23 ± 1.44 mg _GAEs_/g_extract_), and the highest total flavonoid content was found in the ethyl acetate extract of *C. urvillei* subsp. *hayekiana* (25.57 ± 0.51 mg _REs_/g_extract_) [23]. 

Although there are only a few studies that have determined the total phenolic and total flavonoid levels of *Conyza canadensis*, one of these was performed by El Guiche et al. It has been reported that the total phenol content and the total flavonoid contents of *C. canadensis* were found to be 2.54 μg /mg and 5.41 μg/mg, respectively [24]. Our study determined that the total phenol content of *C. canadensis* was 71.34 ± 0.53 mg_GAE_/g_extract_ and the total flavonoid content was 18.91 ± 1.46 mg_CA_/g_extract_, which was similar to the literature. 

In a study investigating the pharmacological activities of *J. fruticans* extracts, the total phenol and total flavonoid contents of the methanol extract were calculated as 82.70 ± 1.84 mg_GAE_/g_extract_ and 70.78 ± 1.86 mg_GAE_/g_extrac_t, respectively [15]. However, the total phenol content of *J. fruticans* in our study was greater than that reported in the previous study, with a total phenol content of 97.41 ± 0.92 mg_GAE_/g_extract_ and total flavonoid content of 19.45 ± 0.84 mg_CA_/g_extract_.

**Figure 1:**
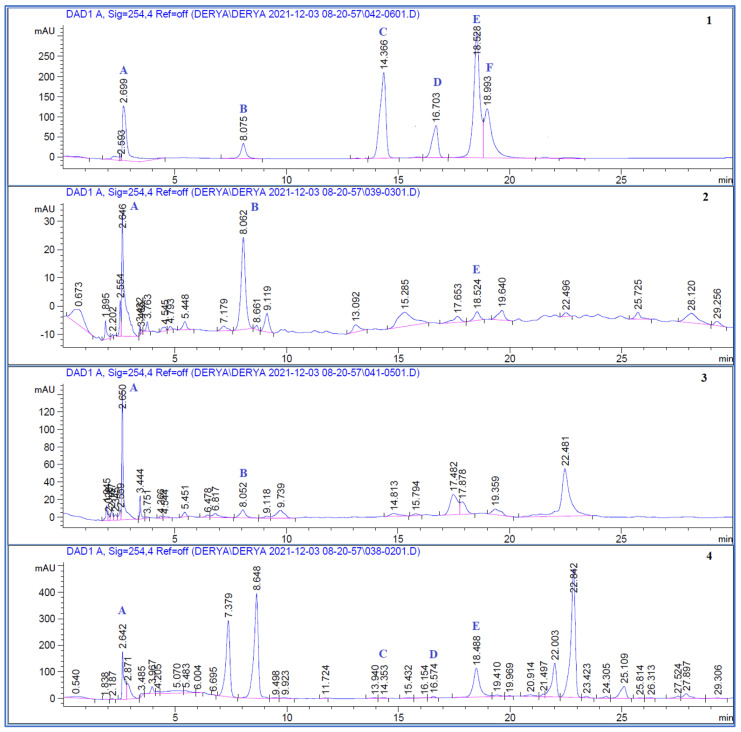
HPLC chromatograms. **1.** Standards: (**A**) Gallic acid, (**B**) Chlorogenic acid, (**C**) *p*-Coumaric acid, (**D**) Ferulic acid, (**E**) Rutin, and (**F**) Hyperoside; **2.** Methanol extract of *Centaurea pichleri* subsp. *pichleri*; **3**. Methanol extract of *Conyza canadensis*; **4**. Methanol extract of *Jasminum fruticans.*

The quantities of chlorogenic acid, *p*-coumaric acid, ferulic acid, gallic acid, hyperoside, and rutin in the plant extracts were calculated (Figure 1). The results and calibration values are shown in Table 1 and Table 2. In addition, the validation values of the HPLC method are given in Table 3 and Table 4.

The highest amount of chlorogenic acid was found in *C. pichleri* subsp. *pichleri* (2.202 ± 0.014%); it could not be found in *J. fruticans. p*-Coumaric acid (0.061 ± 0.007%) and ferulic acid (0.077 ± 0.005%) were detected only in *J. fruticans.* The highest amount of gallic acid (0.271 ± 0.054%) and rutin (0.949 ± 0.008%) were found in *J. fruticans*; however, rutin was not present in *C. canadensis.* Hyperoside was not identified in any of the three extracts (Table 1). A method validation was carried out to prove the accuracy and sustainability of the method.

Reactive oxygen species (ROS) and reactive nitrogen species (RNS) play an essential role in chronic degenerative diseases because they produce oxidative stress in cells. To cope with the oxidative stress created by ROS and RNS, antioxidant defenses need to be strong. Phenolic compounds are known to have antioxidant and anti-inflammatory capacities and many studies have shown that phenolic compounds have a scavenging effect on free radicals [25,26]. In addition, they have been extensively studied in recent years, as they exhibit a wide variety of physiological properties such as anti-mutagenic, anti-carcinogenic, and antimicrobial activity [27,28,29]. It is known that phenolic compounds, such as chlorogenic acid, *p*-coumaric acid, ferulic acid, gallic acid, and rutin, which we detected in the samples in this study, have important antioxidant, anti-inflammatory, anticancer and antimicrobial effects [30,31,32,33,34]. As a result, the quantity of phenolic chemicals in plants is critical. 

The investigation revealed the highest chlorogenic acid amount in *C. pichleri* subsp. *pichleri*. In a previous study with *C. triumfetti* All., chlorogenic acid was found to be high, but gallic acid and rutin were not found [35]. Other research on *C. sivasica* Wagenitz revealed that it contains a significant level of chlorogenic acid; however, gallic acid and rutin were not discovered [36]. Rutin and gallic acid were detected in *C. pichleri* ssp. *pichleri* in our phytochemical analysis in contrast to other studies. 

Abood and Kadhim discovered quercitrin, apigenin, caffeic acid, *p*-coumaric acid, and quercetin in *C. canadensis* [37]. Unlike this study, we detected and quantified chlorogenic acid and gallic acid in *C. canadensis*. 

*p*-Coumaric acid, ferulic acid, gallic acid, and rutin were found in the analysis of the *J. fruticans* extract. Rutin was discovered to be present in greater concentration than the other phenolics. In a previous study with a *J. fruticans* methanol extract; gallic acid, chlorogenic acid, caffeic acid, *p*-coumaric acid, ferulic acid, verbascoside, hyperoside, and rutin were found [15]. However, our research could not confirm the existence of hyperoside and chlorogenic acid. The results found in phytochemical studies may differ due to the place of collection, date of collection, drying method, and extraction methods, even if the same plant is studied [38,39,40].

### 2.2. Antioxidant Activity

**Figure 2:**
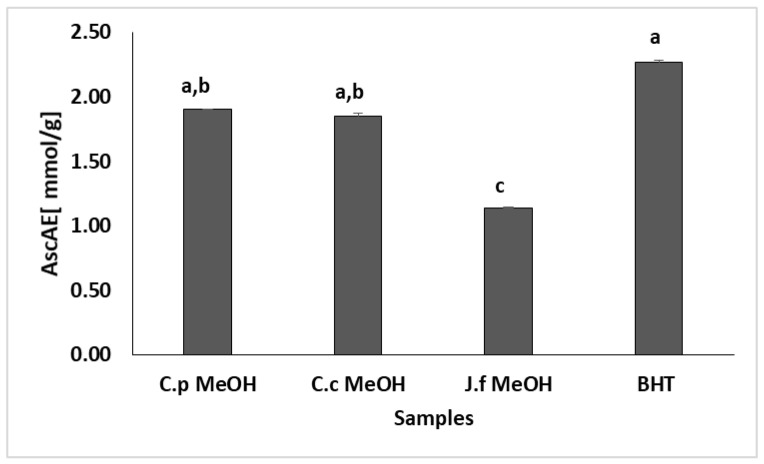
Iron(III) to iron(II) reduction activity of *Centaurea pichleri* subsp. *pichleri, Conyza canadensis,* and *Jasminum fruticans* extracts. Values expressed as mean ± standard error (*n* = 3), statistical analyses by Tukey comparison test. Bars with the same lowercase letters (a–c) are not significantly (*p* > 0.05) different.

DPPH, ABTS, and iron(III) to iron(II) reduction activity assays were used for measuring the antioxidant capacities of the extracts. Antioxidant activities were evaluated by comparing the extracts with an antioxidant standard, and the results are given in Table 5 and Figure 2. 

One of the widely employed techniques used to assess antioxidant capacity is the DPPH scavenging activity test, which is a spectrometric procedure [41]. In our experiments, the inhibition (%) values of the extracts were compared with a positive control and evaluated in terms of antioxidant activity. Concentrations of 4 mg/mL of the three extracts gave an almost close % inhibition value (73.18–77.44%). Although the extracts at the highest concentration studied had the same statistical significance as BHT, they differed in significance as the concentration decreased. At 0.5 mg/mL concentration, *C. pichleri* subsp. *pichleri* was found to be more active than *C. canadensis* and *J. fruticans* with 29.10% inhibition.

Another technique for evaluating antioxidant activity is the ABTS radical scavenging activity test, which detects the radical reduction of samples against this oxidant by employing ABTS as an oxidant agent. The results obtained are given as a Trolox equivalent antioxidant capacity (TEAC) [42]. 

In a previous study investigating the DPPH scavenging effect of four *Centaurea* species (*C. pseudoscabiosa* subsp. *araratica* (Azn.) Wagenitz, *C. pulcherrima* Wild. var. *pulcherrima*, *C. salicifolia* M. Bieb. ex Wild. subsp. *abbreviate*, *C. babylonica* (L.)L.), the highest antioxidant value was found in *C. pulcherrima* var. *pulcherrima* (IC_50_: 187.42 ± 3.11 μg/mL), and the lowest antioxidant activity value was found in *C. pseudoscabiosa* subsp. *araratica* (IC_50_: 670.59 ± 19.43 μg/mL) [43]. The maximum antioxidant value was reported in the *C. canadensis* methanol extract (IC_50_: 120 ± 0.5 μg/mL) in the investigation of Hayet et al. [44]. In the study of El Guiche et al., similar to our results, *C. canadensis*’s antioxidant activity value was found to be 88.19% inhibition [24]. In the antioxidant activity study conducted with *J. fruticans*, the IC_50_ value of the methanol extract was found to be 753.39 ± 15.89 μg/mL [15]. According to our findings, *J. fruticans* has the lowest activity when compared to the other extracts.

The reducing activity from iron(III) to iron(II) is an important investigation in terms of antioxidant activity evaluation and is a technique that demonstrates electron emitting capacity [45]. According to the findings of our investigation, which measured the extracts’ reducing power as ascorbic acid equivalents (AscAE), the *C. pichleri* subsp. *pichleri* (1.852 ± 0.023 AscAE mmol/g) and *C. canadensis* (1.90 ± 0.0006 AscAE mmol/g) extracts had the same significance with BHT (*p* > 0.05). *J. fruticans* (1.141 ± 0.003 AscAE mmol/g) was found to be less active than BHT (2.273 ± 0.01 AscAE mmol/g). No previous assessment of the reducing activity from iron(III) to iron(II) was found in these species.

The antioxidant activities of three different plants were examined in the present study using various methodologies, and it was shown that *C. pichleri* subsp. *pichleri* had slightly more activity than the other species in all antioxidant activity analyses.

### 2.3. Cytotoxic Activity

Prostate cancer is one of the second most common causes of cancer-related death in the USA and Europe, and the incidence of prostate cancer has been increasing worldwide in recent years [46]. Lung cancer is the second most common type of cancer after prostate cancer in men and breast cancer in women. According to 2018 data, lung cancer accounts for 14% of new cancers in men and 13% of new cancers in women in the US [47]. Therefore, it is very important to identify alternative or new drug targets against this type of cancer. In this study, DU145 cell lines, and A549 cell lines were selected as models for these two cancer types. 

**Figure 3:**
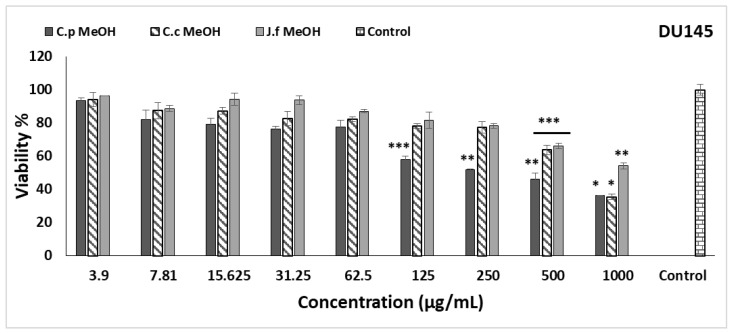
Cytotoxic activity of *Centaurea pichleri* subsp. *pichleri, Conyza canadensis,* and *Jasminum fruticans* extracts on the DU145 cell line. Data are expressed as the mean ± standard error (*n* = 3). Significant differences are indicated as * *p* < 0.001, ** *p* < 0.01, *** *p* < 0.05.

**Figure 4:**
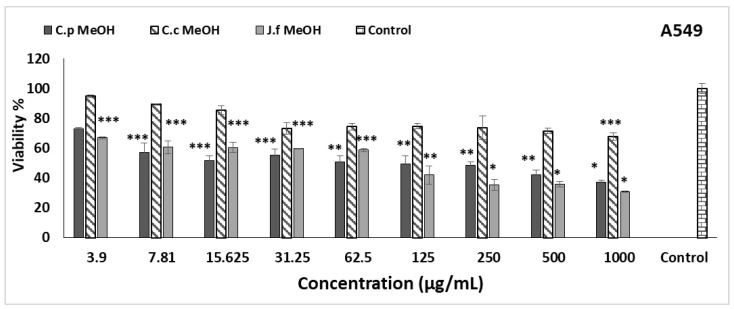
Cytotoxic activity of *Centaurea pichleri* subsp. *pichleri*, *Conyza canadensis*, and *Jasminum fruticans* extracts on the A549 cell line. Data are expressed as the mean ± standard error (*n* = 3). Significant differences are indicated as * *p* < 0.001, ** *p* < 0.01, *** *p* < 0.05.

The effects of three different methanol extracts on DU145, and A549 cell lines were observed using the MTT method. The results are given in Figure 3 and Figure 4. The anti-growth effects of various concentrations of *C. pichleri* subsp. *pichleri*, *C. canadensis*, and *J. fruticans* methanol extracts were compared. *C. pichleri* subsp. *pichleri* methanol extract was found to be more effective at low doses in both cell lines (DU145 and A549 cell lines). In the DU145 cell line, it inhibited the viability by 57.85% at 125 µg/mL concentration, and statistically, *p* < 0.05 was found to be significant compared to the control. In the A549 cell line, even at 7.81 µg/mL concentration, a significant inhibition (*p* < 0.05) of viability was observed compared to the control (57.29%). The cytotoxic IC_50_ value of *C. pichleri* subsp. *pichleri* in the DU145 cell line was 116.26 ± 12.59 µg/mL; however, it was 17.14 ± 2.73 µg/mL in A549. Similarly, *J. fruticans* caused a statistically significant inhibition of viability in the A549 cell line compared to the control at all concentrations studied and the IC_50_ value was found to be 91.97 ± 5.53 µg/mL. Interestingly, only 500 and 1000 µg/mL concentrations were able to significantly inhibit viability in the DU145 cell line. *C. canadensis*, on the other hand, was able to exhibit significant inhibition at high concentrations in both cell lines. It is thought that the fact that the *C. pichleri* subsp. *pichleri* methanol extract is higher than the others in terms of cytotoxic activity may be due to its higher total phenol and total flavonoid contents and its higher antioxidant activity compared to other species. Additionally, it possesses the highest concentration of chlorogenic acid among the three plant extracts, which is likely to contribute to its high activity. Chlorogenic acid is a biologically active polyphenol that also serves as an antioxidant. It has many therapeutic roles, such as antibacterial, hepatoprotective, cardioprotective, anti-inflammatory, neuroprotective, anti-obesity, antiviral, anti-microbial, and anti-hypertensive activity, as well as being a central nervous system (CNS) stimulant [48]. 

The cytotoxic effects of *C. canadensis* have been reported in previous studies. Fractions of *C. canadensis* root and aerial parts were studied, and it was determined that the n-hexane fraction of the roots was active on A549 (IC_50_: 94.73 μg/mL) and H1299 (IC_50_: 84.85 μg/mL) cell lines [13]. According to our results, *C. canadensis* is quite ineffective in the A549 cell line and its IC_50_ level is above 500 μg/mL. In another study, ten different compounds were isolated from *C. canadensis*. It was determined that five of the isolated compounds (conyzapyranone A, conyzapyranone B, 4Z,8Z-matricaria-γ-lactone, 4E,8Z-matricaria-γ-lactone, and spinasterol) were effective in four different cell lines (HeLa, MCF-7, A431, and MRC-5) [49]. Studies of cytotoxic activity on other species have not been found. To the best of our knowledge, a cytotoxicity study has been performed for the first time in these species.

### 2.4. Anti-Inflammatory Activity

Chronic inflammation is influenced by a wide variety of factors, including numerous interleukins, oxygen and nitrogen metabolites, growth factors, and lipid mediators. Many of these are effective not only in inflammation but also in cancer progression. Therefore, it is important to measure these factors [50,51,52]. 

**Figure 5:**
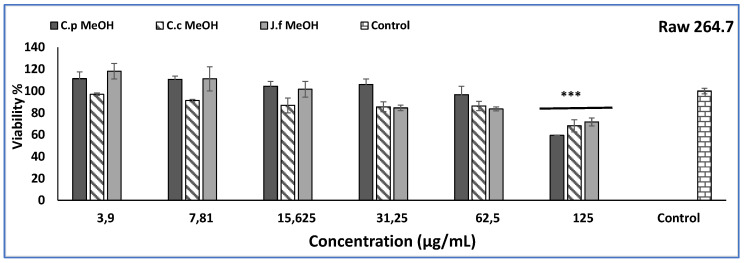
Cytotoxic activity of *Centaurea pichleri* ssp. *pichleri, Conyza canadensis,* and *Jasminum fruticans* extracts on the Raw 264.7 cell line. Data are expressed as the mean ± standard error (*n* = 3). Significant differences are indicated as *** *p* < 0.05.

In this study, the effect of three different methanol extracts on NO inhibition was determined by applying LPS stimulation to Raw 264.7 cells. The toxicity of the extracts was tested before the inflammatory investigation using the MTT technique, and concentrations of 62.5 and 31.25 µg/mL were chosen, where all extracts did not demonstrate significant inhibition compared to the control (Figure 5). *C. pichleri* subsp. *pichleri* methanol extract was found to be more active among all extracts. Although the control group (9.11 ± 2.74 µM) and the LPS group (83.15 ± 4.17 µM) were determined, the *C. pichleri* subsp. *pichleri* methanol extract was found to be significantly lower than the LPS group at both concentrations (47.81 ± 7.15 and 38.48 ± 3.62 µM). Another parameter of anti-inflammatory studies is to evaluate the effects on cytokines. Cytokines play an important role, especially in chronic inflammatory diseases [53,54]. Likewise, when TNF-α, IFN-γ, and PGE_2_ levels were examined, it was found that the *C. pichleri* subsp. *pichleri* methanol extract was more active than the others. The TNF-α, PGE_2_, and IFNƔ value of the *C. pichleri* subsp. *pichleri* methanol extract (62.5 µg/mL) was found to be 1953.57 ± 21.48 pg/mL, 1854.17 ± 9.47 pg/mL, and 99.1 ± 3.65 pg/mL, respectively. The results found were evaluated as significant compared to the control groups (Table 6). To the best of our knowledge, this is the first study in which anti-inflammatory values (TNF-α, IFN-γ, PGE_2_ levels, and NO amount) are given together.

## 3. Materials and Method

### 3.1. Plant Material

Specimens of *Centaurea pichleri* subsp. *pichleri* and *Jasminum fruticans* were collected from Ankara-Beynam in 2021. The *Conyza canadensis* specimen was collected from Balıkesir-Altınoluk, in 2020. The voucher samples of *J. fruticans*, *C. pichleri* subsp. *pichleri*, and *C. canadensis* were deposited in the Herbarium of Ankara University, Faculty of Pharmacy AEF 30773, AEF 30961, and AEF30963, respectively. 

### 3.2. Sample Preparation

Dried samples were powdered and extracted with methanol (99.9%, Riedel-De-Haën), five times at room temperature. The extraction was finished using an ultrasonic bath (60 min.) (ISOLAB 621.05.010) [55]. After being filtered, the extracts were concentrated with an evaporator (Heidolph WB2000).

### 3.3. Chemical Analysis

#### 3.3.1. Determination of Total Phenolic Content

To determine the total amount of phenolic content, the extracts were mixed with distilled water (3.95 mL), Folin-Ciocalteu reagent (250 µL), and 20% Na_2_CO_3_ (750 µL), respectively. After the samples were kept at room temperature (25 °C) for 2 h, measurements were made at 760 nm. Gallic acid was used as a standard [56].

#### 3.3.2. Determination of Total Flavonoid Content

To determine the total amount of flavonoids, 4 mL of distilled water and 0.3 mL of 5% NaNO_2_ were added to the extracts with the concentration adjusted. After waiting for 5 min, 0.3 mL of 10% AlCl_3_6H_2_O solution was added. Then, 2 mL of 1 M NaOH was added to the samples, resulting in a total volume of 10 mL. Measurement of the samples was made at 510 nm using the Shimadzu Spectrophotometer UV 1800 (Washington, USA). Catechin was used as the reference substance [57].

#### 3.3.3. High Performance Liquid Chromatography (HPLC) Analysis

Sample solutions were prepared at a concentration of 4 mg/mL. Stock standard solutions (chlorogenic acid, coumaric acid, ferulic acid, gallic acid, hyperoside, and rutin) were prepared at 500 μg/mL concentration. A Waters Spherisorb C_18_ column (25 cm × 4.6 mm, 5 μm) was used for the HPLC analysis. The mobile phase consisted of 0.01% formic acid (A) and acetonitrile (B) in the gradient system. The flow rate was 1 mL/min, the column temperature was 40 °C and all analyses were carried out at the 254 nm wavelength.

Five different concentrations of standards were injected in triplicate. For quantification, the calibration curve of each standard was obtained. For method validation, accuracy, precision, the limit of detection (LOD), the limit of quantitation (LOQ), and recovery values were calculated for method validation [39,41]. The precision of the method (intra-day and inter-day variation) was carried out, and the differences were expressed by the relative standard deviation (RSD). For analysis of the LOD and LOQ values, 10 injections of standards were made, and the signal/noise value calculated. The LOD signal/noise value was 3:1, while the LOQ signal/noise value was 10:1. For the recovery analysis, 3 different known concentrations of the standard were added to the sample and the recovery percentage was calculated. For the robustness analysis, minor changes in flow rate, column temperature, mobile phase, and wavelength were made and checked to affect the analysis.

### 3.4. Antioxidant Activity

#### 3.4.1. DPPH^●^ Radical Scavenging Activity

The Gyamfi et al. method was used to calculate the DPPH^●^ radical scavenging effects of the samples [58]. The samples were mixed with a Tris-HCl buffer (50 nM, pH 7.4) and a DPPH solution (0.1 mM) was prepared in methanol. A Butylated Hydroxy Toluene (BHT) standard antioxidant was used as a positive control. After the samples were incubated in the dark for 30 min at room temperature, measurements were made at 517 nm. The process was repeated three times in parallel and the inhibition % calculations were made using the equation below:

Inhibition % = [(Abs_control_ − Abs_sample_)/Abs_control_] × 100

#### 3.4.2. ABTS^●+^ Radical Scavenging Activity

The Re et al. method was used to calculate the ABTS^●+^ radical scavenging effects of the samples [59]. A solution of ABTS+ radical (7 mM) with the absorbance adjusted to 0.700 (±0.030) at 734 nm was obtained for analysis. The reaction kinetics were measured and recorded at 734 nm at 1 min periods for 30 min using 990 μL of the prepared radical solution and 10 μL of the samples. Trolox equivalents were calculated as percentages of inhibition measured (TEAC). BHT standard antioxidant was used as a positive control. The process was repeated three times in parallel.

#### 3.4.3. Iron(III) to iron(II) reduction activity (FRAP)

The Koşar et al. method was used to calculate the ability of the samples to reduce iron(III) [60]. The sample solutions (1 mL) were mixed with 2.5 mL of a 0.2 M phosphate buffer (pH 6.6) and 2.5 mL of 1% (*w*/*v*) potassium hexacyanoferrate solution (K_3_[Fe(CN)_6_]^+3^). After 30 min of incubation at 50 °C, 2.5 mL of 10% (*w*/*v*) trichloroacetic acid (TCA) was added and centrifuged for 10 min. It was then mixed with 2.5 mL of H_2_O and 0.5 mL of 0.1% (*w*/*v*) Iron(III) chloride (FeCl_3_). The absorbances of the samples were measured at 700 nm. The reducing activities of the extracts were expressed as ascorbic acid equivalents (AscAE) per mmol ascorbic acid/g sample.

### 3.5. Cytotoxic Activity

#### 3.5.1. Cell Lines and Cell Culture Methods

The DU145 human prostate cancer cell line and A549 human lung cancer cell line were purchased from American Type Culture Collection (ATCC HTB-81^™^, ATCC CCL-185, Manassas, VA, USA) and maintained in Eagle’s Minimum Essential Medium (EMEM) and RPMI and supplemented with 10% fetal bovine serum (FBS), and 100 μg/L penicillin/streptomycin, at 37 °C in a 5% CO_2_ atmosphere.

#### 3.5.2. Determination of Cell Viability Assay

The cytotoxic activity of the extracts on A549 and DU145 cells was determined by the MTT (3-[4, 5-dimethylthiazole-2-yl]-2, 5-diphenyltetrazolium bromide) colorimetric method. Then, 24 h before the study, the cells were inoculated in a 96-well microplate with 1 × 10^4^ cells at 100 µL per well and incubated. After 24 h, the medium on the cells adhered to the plate was discarded. A stock solution of 1 mg/mL of the extracts to be applied was prepared. It was then prepared by diluting it in the medium at concentrations of 3.9 μg/mL, 7.81 μg/mL, 15.6 μg/mL, 31.25 μg/mL, 62.5 μg/mL, 125 μg/mL, 250 μg/mL, 500 μg/mL, and 1000 μg/mL. A total of 100 μL of each sample was added to the well. After 24 h of incubation, the medium in the wells was emptied. The wells were then filled with 100 µL of the MTT solution diluted 1/10 with a medium made from 5 mg/mL (in PBS) MTT stock. After keeping the plates in a CO_2_ incubator at 37 °C for 4 h, 100 µL of DMSO was added to each well and the formazan crystals formed by the MTT were dissolved. After 10 min, each well was read using a microplate reader at the 540 nm wavelength [61]:

% Viability = [(Abss_ample_ × 100)/Abs_control_]

### 3.6. Anti-Inflammatory Activity

The murine macrophage Raw 264.7 cell line was obtained from the American Type Culture Collection (ATCC TIB-71, Manassas, VA, USA) and kept at 37 °C in a 5% CO_2_ environment in Dulbecco’s Modified Eagle’s medium (DMEM) supplemented with 10% FBS, and 100 µg/L penicillin/streptomycin. Using the aforementioned MTT assay, the toxicity of the extracts in the Raw 264.7 cell line at concentrations between 3.9 and 125 µg/mL was assessed. For anti-inflammatory activity, the RAW 264.7 cells were seeded at a number of 5 × 10^5^ in six-well plates and incubated for 24 h at 37 °C and 5% CO_2_. After incubation, 1 µg/mL lipopolysaccharide (LPS) was added to all other wells except the control well. The extracts were applied to the wells at 31.25 and 62.5 µg/mL concentrations, respectively. Supernatants were collected and centrifuged after 24 h (10 min., 700× *g*).

For nitric oxide (NO) determination, 50 µL of Griess reagent (0.1% N-(1-naphthyl)-ethylene diamine, 1% sulfanilamide in 5% phosphoric acid) was mixed with equal volumes of culture supernatants, respectively. After 10 min of incubation at room temperature, the absorbance was measured at 550 nm. The calibration curve’s standard was prepared using sodium nitrite [62].

PGE2 (Andygene AD1630Mo), IFN-Ɣ(Andygene AD2783Mo), and TNF-α (Andygene AD2726Mo) cytokine levels were determined using the commercial kit technique with supernatant collected from the wells.

### 3.7. Statistical Analysis

Analysis was performed in at least triplicate and the mean values were calculated. All data were presented as the mean ± standard deviation (SD), and relative standard deviation (RSD). All these calculations were made using the Microsoft Excel program.

The Levene test was used to evaluate the variance homogeneity. One-way analysis of variance was used for comparisons between more than two groups. The Dunnett T3 test and Tukey’s test were used for multiple comparisons. The data were evaluated with SPSS Version 11.0 statistic software package. The significance level was set at *p* < 0.05.

## 4. Conclusions

In this study, three different traditional plant methanol extracts were evaluated in terms of pharmacological activities. Some activities in this study were performed for the first time on these species. In addition, to the best of our knowledge, this is the first report to show the pharmacological activity potentials and some phenolic compound contents of the three traditionally used plants together. In this study, previous studies on these plants were collected and a collective evaluation of the three plants was made in terms of pharmacological activity with new activity studies. Antioxidant activity (DPPH, ABTS, and iron(III) to iron(II) reduction), cytotoxic activity (DU145 and A549), and anti-inflammatory activity (NO amount, TNF-α, IFN-γ, PGE_2_ levels) were evaluated, and phytochemical analyses were carried out by HPLC. The *C. pichleri* ssp. *pichleri* methanol extract, which is rich in total phenols and total flavonoids and has high antioxidant activity, was found to be effective in terms of both cytotoxic activity and anti-inflammatory activity. The *J. fruticans* methanol extract has higher total phenol and total flavonoid contents than the *C. canadensis* methanol extract, but it was found to be less active in terms of antioxidant and anti-inflammatory activity and cytotoxicity. This is considered to be caused by the various active substances they each possess. So, this study is an important step for future studies. However, more studies may result in the discovery and development of innovative medications for the pharmaceutical industry. These results will aid in the creation of pharmacologically active natural products, and functional dietary supplements for the prevention of various diseases. The fractionation, isolation of active ingredients, and identification of the mechanisms underlying these pharmacological activities will also illuminate the traditional uses of these plants.

## Figures and Tables

**Table 1:** Total phenol content, total flavonoid content, and quantitative determination of chlorogenic acid, coumaric acid, ferulic acid, gallic acid, hyperoside, and rutin in the methanol extracts.

	Methanol Extract of *Centaurea pichleri* subsp. *pichleri*	Methanol Extract of *Conyza canadensis*	Methanol Extract of *Jasminum fruticans*
Total Phenol (mg_GAE_/g_extract_)	98.19 ± 1.64	71.34 ± 0.53	97.41 ± 0.92
Total Flavonoid (mg_CA_/g_extract_)	21.85 ± 0.64	18.91 ± 1.46	19.45 ± 0.84
Chlorogenic acid (% ± SD **)	2.202 ± 0.014	1.110 ± 0.011	ND *
*p*-Coumaric acid (% ± SD **)	ND *	ND *	0.061 ± 0.007
Ferulic acid (% ± SD **)	ND *	ND *	0.077 ± 0.005
Gallic acid (% ± SD **)	0.031 ± 0.002	0.249 ± 0.101	0.271 ± 0.054
Hyperoside (% ± SD **)	ND *	ND *	ND *
Rutin (% ± SD **)	0.049 ± 0.002	ND *	0.949 ± 0.008

* ND: Not Detected; ** SD: Standard Deviation.

**Table 2:** Calibration values for standards.

	Calibration Range (μg/mL)	Linear Equation	Correlation Factor (r^2^ ± SD *)	LOD (μg/mL)	LOQ (μg/mL)
Chlorogenic acid	25–250	y = 7.2394 x − 167.78	0.993 ± 0.002	0.006	0.020
*p*-Coumaric acid	50–500	y = 21.376 x + 206.94	0.999 ± 0.0005	0.0008	0.002
Ferulic acid	50–500	y = 18.588 x + 5.3289	0.992 ± 0.001	0.0003	0.001
Gallic acid	50–500	y = 44.783 x + 109.09	0.998 ± 0.001	0.00003	0.0001
Hyperoside	50–500	y = 34.63 x + 636.51	0.992 ± 0.002	0.00005	0.0001
Rutin	50–500	y = 58.596 x − 61.545	0.9952 ± 0.002	0.00002	0.00007

* SD: Standard Deviation.

**Table 3:** Precision data of the method.

	Amount (μg/mL)	Intra-Day Precision (RSD * %)	Inter-Day Precision (RSD * %)
Chlorogenic acid	50 200 500	0.704 0.616 0.046	0.295 0.006 0.651
*p*-Coumaric acid	50 200 500	1.367 0.069 0.142	0.311 1.203 0.651
Ferulic acid	50 200 500	0.570 0.046 0.094	0.132 0.532 0.321
Gallic acid	25 100 250	0.733 2.710 2.149	2.007 3.189 2.493
Hyperoside	50 200 500	1.559 0.807 0.208	3.137 1.068 0.452
Rutin	50 200 500	0.076 0.622 0.011	0.352 0.878 0.453

* RSD: Relative Standard Deviation.

**Table 4:** Recovery assay’s statistical data of the method (*n* = 3).

Standards	Concentration in Sample (mg/mL)	Amount Spiked (mg/mL)	Mean Amount Found in the Mixture (mg/mL)	Mean Recovery (% ± SD *)	RSD ** (%)
Chlorogenic acid	0.08	0.04 0.08 0.16	0.06 0.08 0.12	103.771 ± 0.719 103.224 ± 1.055 98.243 ± 2.726	1.753 1.159 2.774
*p*-Coumaric acid	0.002	0.001 0.002 0.004	0.0015 0.002 0.003	104.073 ± 2.039 101.827 ± 2.219 101.437 ± 2.657	1.959 2.179 2.619
Ferulic acid	0.003	0.0015 0.003 0.006	0.00375 0.003 0.0045	102.858 ± 2.357 102.396 ± 1.797 102.446 ± 2.448	2.292 1.755 2.389
Gallic acid	0.01	0.005 0.01 0.02	0.0075 0.01 0.015	97.996 ± 2.301 96.539 ± 1.280 98.900 ± 2.254	2.348 1.326 2.279
Rutin	0.03	0.015 0.03 0.06	0.0225 0.03 0.045	103.019 ± 1.806 102.549 ± 1.189 102.946 ± 1.906	1.753 1.159 1.852

* SD: Standard Deviation, ** RSD: Relative Standard Deviation.

**Table 5:** DPPH and ABTS radical scavenging activities of *Centaurea pichleri* subsp. *pichleri*, *Conyza canadensis*, and *Jasminum fruticans* extracts.

	*Centaurea pichleri* subsp. *pichleri*		*Conyza canadensis*		*Jasminum fruticans*		BHT	
Conc. (mg/mL)	DPPH Inhibition (%)	ABTS TEAC mmol/L/Trolox	DPPH Inhibition (%)	ABTS TEAC mmol/L/Trolox	DPPH Inhibition (%)	ABTS TEAC mmol/L/Trolox	DPPH Inhibition (%)	ABTS TEAC mmol/L/Trolox
4	77.11 ± 0.82 ^a^	2.56 ± 0.04 *	77.44 ± 0.24 ^a^	2.56 ± 0.005 *	73.18 ± 0.15 ^a,b^	2.57 ± 0.03 *	84.19 ± 1.27 ^a^	2.58 ± 0.01 *
2	73.42 ± 0.54 ^a,b^	2.55 ± 0.0 *5	76.43 ± 3.92 ^a,b^	2.56 ± 0.03 *	53.67 ± 0.99 ^b^	2.56 ± 0.003 *	84.01 ± 0.8 ^a^	2.57 ± 0.03 *
1	58.37 ± 2.90 ^b^	2.49 ± 0.0 *3	43.12 ± 1.87 ^c^	2.26 ± 0.13 **	26.40 ± 2.18 ^d^	2.35 ± 0.15 **	83.92 ± 2.14 ^a^	2.56 ± 0.12 *
0.5	29.10 ± 3.33 ^d^	1.66 ± 0.06 ***	20.36 ± 0.29 ^d,e^	1.44 ± 0.07 ***	11.72 ± 0.45 ^e^	1.59 ± 0.11 ***	82.17 ± 4.13 ^a^	2.56 ± 0.08 *

Values expressed as mean ± standard error (*n* = 3), statistical analyses by Tukey comparison test. Bars with the same lower-case letters (a–e), and symbols (*–***) are not significantly (*p* > 0.05) different.

**Table 6:** The effect of the extracts on TNF-α, IFN-γ, PGE_2_ levels, and NO amount.

	TNF-α (pg/mL)		PGE_2_ (pg/mL)		IFNƔ (pg/mL)		NO (µM)	
	31.25 µg/mL	62.5 µg/mL	31.25 µg/mL	62.5 µg/mL	31.25 µg/mL	62.5 µg/mL	31.25 µg/mL	62.5 µg/mL
*Centaurea pichleri* subsp. *pichleri*	2009.12 ± 18.96 ***	1953.57 ± 21.48 **	1922.73 ± 6.48 **	1854.17 ± 9.47 **	112.41 ± 9.14 ***	99.1 ± 3.65 **	47.81 ± 7.15 **	38.48 ± 3.62 **
*Conyza canadensis*	2451.29 ± 18.19	2312.42 ± 25.53	2315.54 ± 21.47	2259.89 ± 17.82	151.24 ± 3.75	132.14 ± 5.58	75.47 ± 8.14	67.64 ± 5.12
*Jasminum fruticans*	2217.191 ± 11.58 ***	2109.82 ± 9.56 ***	2157.87 ± 25.89 ***	2121.62 ± 22.49 ***	126.45 ± 7.59	115.63 ± 8.74 ***	61.91 ± 4.18 ***	55.72 ± 2.41 ***
Control		986.4 ± 8.94 *		1017.16 ± 18.79 *		48.51 ± 4.72 *		9.11 ± 2.74 *
LPS group		2517.73 ± 6.55		2416.72 ± 9.57		157.78 ± 9.63		83.15 ± 4.17

Anti-inflammatory activity of *Centaurea pichleri* subsp. *pichleri*, *Conyza canadensis*, and *Jasminum fruticans* extracts on the Raw 264.7 cell line. Data are expressed as the mean ± standard error (*n* = 3). Significant differences are indicated as * *p* < 0.001, ** *p* < 0.01, *** *p* < 0.05.

## Data Availability

The data presented in this study are available in the article.
